# The Impact of Computed Tomography Measurements of Sarcopenia on Postoperative and Oncologic Outcomes in Patients Undergoing Cytoreductive Surgery and Hyperthermic Intraperitoneal Chemotherapy

**DOI:** 10.3390/curroncol29120730

**Published:** 2022-11-29

**Authors:** Maher Al Khaldi, Massine Fellouah, Pierre Drolet, Julien Côté, Bertrand Trilling, Alexandre Brind’Amour, Alexandre Dugas, Jean-François Tremblay, Suzanne Fortin, Lara De Guerké, Marie-Hélène Auclair, Pierre Dubé, Mikaël Soucisse, Lucas Sideris

**Affiliations:** 1 Division of Surgical Oncology, Department of Surgery, Hôpital Maisonneuve-Rosemont, CIUSS de l’Est de l’ïle de Montréal, Montréal, QC H1T 2M4, Canada; 2 Department of Anesthesiology, Hôpital Maisonneuve-Rosemont, CIUSSS de l’Est de l’Île de Montréal, Montréal, QC H1T 2M4, Canada; 3 Division of Surgical Oncology, Department of Surgery, CHU de Québec-Université Laval, Québec, QC G1R 2J6, Canada; 4 Division Abdominal Radiology, Department of Radiology, Maisonneuve-Rosemont, CIUSSS de l’Est de l’Île de Montréal, Montréal, QC H1T 2M4, Canada; 5 Division of Colorectal Surgery, Department of Surgery, Hôpital Maisonneuve-Rosemont, CIUSSS de l’Est de l’Île de Montréal, Montréal, QC H1T 2M4, Canada; 6 Division of Gynecologic Oncology, Department of Surgery, Hôpital Maisonneuve-Rosemont, CIUSSS de l’Est de l’Île de Montréal, Montréal, QC H1T 2M4, Canada

**Keywords:** sarcopenia, peritoneal metastases, cytoreductive surgery and hyperthermic intraperitoneal chemotherapy

## Abstract

Cytoreductive surgery with hyperthermic intraperitoneal chemotherapy (CRS-HIPEC) is a treatment option for peritoneal metastases (PM) but is associated with significant postoperative morbidity. The aim of this study was to determine the prognostic value of computed tomographic (CT)-measured sarcopenia on postoperative outcomes and survival in patients undergoing CRS-HIPEC for PM from various origins. A retrospective cohort study was conducted between 2012 and 2020. Three-hundred and twelve patients (mean age 57.6 ± 10.3, 34.3% male) were included, of which 88 (28.2%) were sarcopenic. PM from a colorectal origin was the most common in both groups. The proportion of major postoperative complications (Clavien-Dindo ≥ III) was not higher in the sarcopenic group (15.9% in sarcopenic patients vs. 23.2% in nonsarcopenic patients, *p* = 0.17). The mean Comprehensive Complication Index scores, HIPEC-related toxicities, length of hospital stay, and duration of parenteral nutrition were comparable regardless of sarcopenia status. In the multivariate logistic regression analysis of severe complications, only peritoneal carcinomatosis index reached statistical significance (OR, 1.05; 95% CI, 1.01 to 1.08, *p* = 0.007). Sarcopenia did not impact origin-specific overall survival on Cox regression analysis. Sarcopenia was not associated with worse rates of postoperative severe complications or worse survival rates. Future prospective studies are required before considering sarcopenia as part of preoperative risk assessment.

## 1. Introduction

Determining the optimal care of surgical patients includes understanding their risk factors for postoperative complications and mortality. Developing objective techniques that aid clinicians at screening the patients at greater risk of surgical morbidity is crucial, especially for major surgeries. Sarcopenia, defined as the loss of skeletal muscle mass and strength, is associated with worse outcomes in both surgical and nonsurgical patients [1]. The development of such muscle mass depletion is multifactorial, and include patient malnutrition, age, functional status, hereditary causes, chemotherapy, ongoing systemic inflammatory states, and patient comorbidities such as cancer [1,2]. Sarcopenia has been specifically studied as a predictor of oncologic surgery outcomes (i.e., morbidity, mortality, and overall survival) in various types of cancer, including but not limited to colorectal [3], liver [4,5,6] and pancreatic cancers [7].

Cross-sectional imaging techniques, such as computer tomography (CT) scans, are routinely performed prior to surgery for cancer staging and operative planning. An image-based definition of sarcopenia has been previously established [8] and is now widely used across studies [9]. Briefly, a skeletal muscle index (SMI) is generated by calculating the total muscle area (TMA) at an axial image through the third lumbar vertebrae (L3) and normalizing it to patient stature. SMI Sex-specific cutoff values are used to classify patients as sarcopenic or not [8].

Cytoreductive surgery combined with hyperthermic intraperitoneal chemotherapy (CRS-HIPEC) is increasingly used for the treatment of malignant peritoneal diseases across the world [10]. However, it is associated with significant postoperative complications and mortality [11,12], especially in the elderly patients [13]. Severe postoperative complications associated with CRS-HIPEC could reach 30% and mortality rates approach 3% [14]. The impact of preoperative sarcopenia on postoperative outcomes in patients undergoing CRS-HIPEC for malignant mesothelioma and pseudomyxoma peritonei [15] as well as colorectal cancer [16,17,18,19] has been studied. However, due to the paucity of studies and their inconsistent conclusions, the impact of sarcopenia on postoperative morbidity and outcomes for patients undergoing CRS-HIPEC remains inconclusive.

In this study, we investigated the prognostic effect of computed tomographic (CT)-measured sarcopenia on postoperative outcomes and survival in patients undergoing CRS-HIPEC for peritoneal metastases (PM) from various origins.

## 2. Materials and Methods

Design and Setting: After approval from the institutional research ethics board (no. 2021-2326, 8 July 2020), we conducted a retrospective cohort study at the Maisonneuve-Rosemont Hospital (MRH) evaluating the impact of sarcopenia on postoperative outcomes at 90 days and survival between 2012 and 2020. All consecutive patients were included in the study. The technique for delivering HIPEC has transitioned from “open” to “closed” at our institution in January 2012. We aimed for a more homogeneous surgical population by including patients from that date onward. The MRH is a tertiary referral center with expertise in the management of patients with peritoneal malignancies.

Patient selection: Adult patients (≥18 years) who were diagnosed with synchronous or metachronous PM from different origins and who underwent CRS-HIPEC with a completeness of cytoreduction (CC) score of 0, 1 or 2 were included. Exclusion criteria were the following: missing preoperative CT scan for SMI calculation, missing height for SMI calculation, and patients who underwent CRS-HIPEC with a CC-3 score.

Surgical intervention: Prior to surgery, a full metastatic workup with appropriate imaging was performed for each patient. During surgical intervention, a comprehensive exploration of the abdomen was performed, and a peritoneal cancer index (PCI) was calculated [20]. A score of CC-0 indicates no residual peritoneal disease following CRS. A score of CC-1 represents residual disease < 2.5 mm, CC-2 indicates residual tumor between 2.5 mm and 2.5 cm, and CC-3 indicates residual tumor > 2.5 cm). Cytoreductive surgery was performed followed by administration of HIPEC using the closed abdomen technique at a targeted temperature of 42 °C. For patients with colorectal or appendiceal PM, HIPEC was only performed with CC-0 or CC-1 scores. HIPEC was also performed on patients with ovarian PM or mesothelioma with CC-2 scores. The chemotherapy agent used varied according to the origin of PM. The duration of HIPEC varied from 30 to 90 min depending on the type of drug used. Mitomycin C (35 mg/m^2^/L) for 90 min or Oxaliplatin (460 mg/m^2^) for 30 min were used for nongynecological PM. Concomitant infusion of 5-Fluorouracil (400 mg/m^2^)/Leucovorin (20 mg/m^2^) was generally administered for patients receiving Oxaliplatin. Carboplatin (at a dose of 800 to 900 mg until 2017, with a protocol amendment for an area under the curve of 10, averaging a dose of 1000 mg) for 90 min or Cisplatin (100 mg/m^2^) for 60 min were used for gynecological PM.

**Figure 1:**
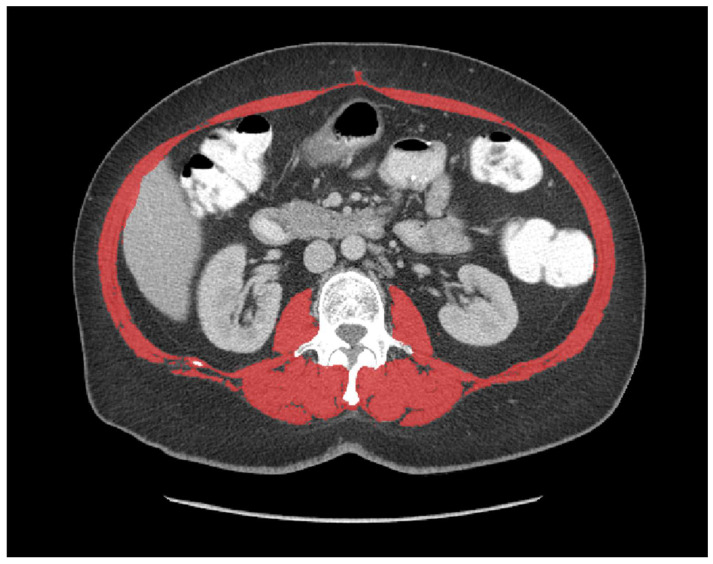
Muscle surface area measurement using an axial slice at L3. Muscles targeted included psoas, erector spinae, quadratus lumborum, transversus abdominus, external and internal obliques, and rectus abdominus muscles using a threshold of −29 to +150 HU (red).

CT-measured sarcopenia using the skeletal muscle index: Preoperative abdominal CT scans were extracted in DICOM file format for further analysis using the CoreSlicer web-based software tool (https://coreslicer.com/ accessed between July 2019 to January 2020 version 1.0.0, Montreal, Quebec) which enables specific tissue demarcation using Hounsfield unit (HU) thresholds. Cross-sectional surface measurements of muscle tissue (psoas, erector spinae, quadratus lumborum, transversus abdominus, external and internal obliques, and rectus abdominus), at the level of L3 were performed by a radiologist-trained assessor blinded to patient outcomes. Muscle-specific units used were −29 to +150 HU [21] and skeletal muscle was demarcated in an automatic manner (Figure 1). Manual corrections were performed as necessary. The SMI (cm^2^/m^2^) was calculated by dividing the total cross-sectional muscle area (cm^2^) by patient height (m^2^). Sarcopenic patients were identified as having SMI of <52.4 cm^2^/m^2^ for men and <38.5 cm^2^/m^2^ for women [8].

Variables and definitions. For each patient, basic demographic information included age at time of surgery, sex, weight, height, skeletal muscle surface area at L3, origin of PM as well as timing of PM. Synchronous PM referred to cases in which peritoneal disease was identified at the same time as the primary tumour. Metachronous PM was defined as disease occurring >6 months of primary diagnosis. Primary PM referred to epithelial or multicystic mesothelioma. Intraoperative variables were operative time, estimated blood loss, CC score and intraoperative PCI. HIPEC-related toxicities were recorded and encompassed hematologic, metabolic, neurologic, and gastrointestinal adverse effects. Postoperative parental nutrition duration, length of hospital stay, and perioperative death were recorded. All postoperative complications at 90 days were categorized according to the Clavien-Dindo (CD) score [22] and the Comprehensive Complication Index (CCI) [23]. Severe complications were defined as CD ≥ III. CCI scores were obtained with an EXCEL sheet with integrated CCI^®^ formula (https://www.assessurgery.com/about_cci-calculator/ accessed on 22 May 2021). Potential confounders of the association between sarcopenia and outcomes were selected before conducting any analyses and included sex, age, body mass index (BMI), intraoperative PCI, and blood loss.

Statistical analysis. Patient baseline and perioperative characteristics are reported based on the presence or absence of sarcopenia. These characteristics were compared using Chi-square, Fisher’s or unpaired *T* tests. Since assessing the relation between sarcopenia and the occurrence of severe postoperative adverse events was the primary outcome, sarcopenic vs. nonsarcopenic patients were compared using Fisher’s exact test for the development of Clavien-Dindo grade III or higher complications. Furthermore, multiple logistic regression was used to assess the effect of age, sex, BMI, PCI and surgical blood loss, alongside the presence or absence of sarcopenia, on the occurrence of Clavien-Dindo grade III or higher complications. Since the secondary outcome of the study was to assess the impact of sarcopenia on overall survival, we used simple Kaplan–Meier survival curves followed by the logrank (Mantel-Cox) test to compare sarcopenic vs. nonsarcopenic patients. We further assessed the impact of sarcopenia on survival in the presence of potential confounders with Cox proportional hazards regression model. Age, sex, BMI, PCI and origin of the carcinomatosis were entered alongside the existence of sarcopenia or not in the regression model. These variables were chosen because they have been shown to be pre-intervention factors related to survival following cytoreductive surgery and hyperthermic intraperitoneal chemotherapy in previous studies [24,25]. Analyses were conducted with Prism 9.3 (GraphPad Software, San Diego, CA, USA).

## 3. Results

### 3.1. Patient Characteristics

**Figure 2:**
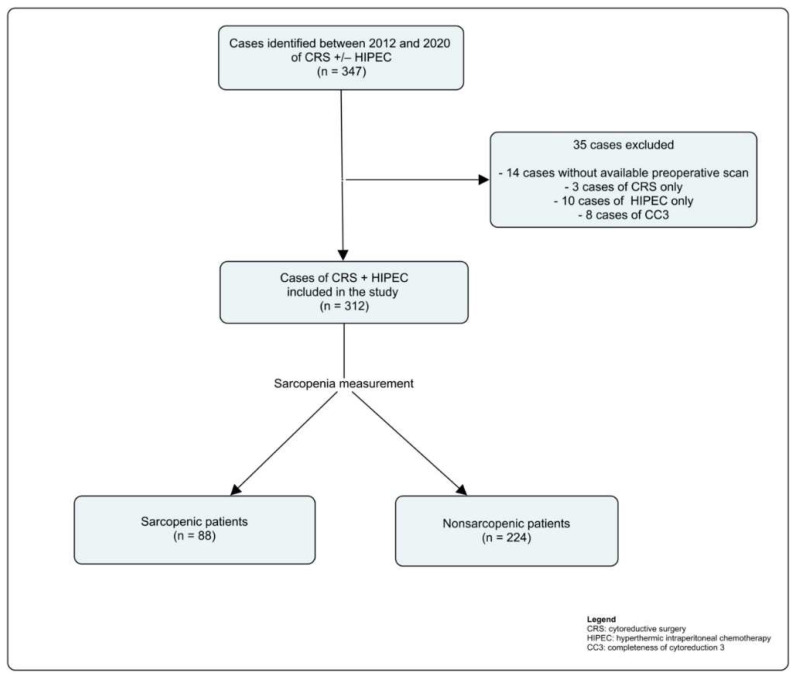
Flow chart of patient inclusion and exclusion.

Among 347 patients who underwent CRS ± HIPEC between 2012 and 2020, 312 met the criteria for analysis (Figure 2). Thirty-five patients were excluded from the study: 14 had no available preoperative scan for SMI calculation, 3 underwent CRS only, 10 underwent prophylactic HIPEC only and 8 had a CC-3 score. Of the 312 patients included, 88 (28.2%) were sarcopenic (Table 1). Overall, there were more females than males (65.7% vs. 34.3%, respectively). The proportion of males in the sarcopenic group was significantly higher than in the nonsarcopenic group (46.6% vs. 29.5%, *p* = 0.0053). The mean delay between the day of surgery and preoperative scan was comparable in both groups (42.1 days for sarcopenic patients vs. 45.0 for nonsarcopenic patients, *p* = 0.53). Sarcopenic patients had lower mean BMI (23.6 kg/m^2^ vs. 27.7 kg/m^2^, *p* ≤0.0001) and a lower mean SMI (41.4 cm^2^/m^2^ vs. 51.2 cm^2^/m^2^, *p* < 0.0001). The distribution of the origins of PM was similar between both groups, with PM from colorectal origin being the most common. Most cases of PM presented synchronously.

### 3.2. Postoperative Outcomes

Mean operative time, estimated blood loss, distribution of CC score, and intraoperative PCI were similar in both groups (Table 2). Major postoperative complications occurred in 66/312 patients (21.2%) and were not higher in the sarcopenic group (15.9% in sarcopenic patients vs. 23.2% in nonsarcopenic patients, *p* = 0.17). The CCI score, all HIPEC-related toxicities, length of hospital stay, and duration of parenteral nutrition were comparable regardless of sarcopenia status. Overall perioperative mortality (CD V) occurred in 3 patients (1.0%) and was similar between sarcopenic and nonsarcopenic patients (1.1% vs. 0.9%, respectively, *p* = 0.84). A subanalysis of postoperative outcomes stratified by sarcopenic state and PM origin was performed. No differences were noticed for all origins, regardless or sarcopenia status (Supplementary Table S1).

### 3.3. Multivariate Analysis and Survival

**Figure 3:**
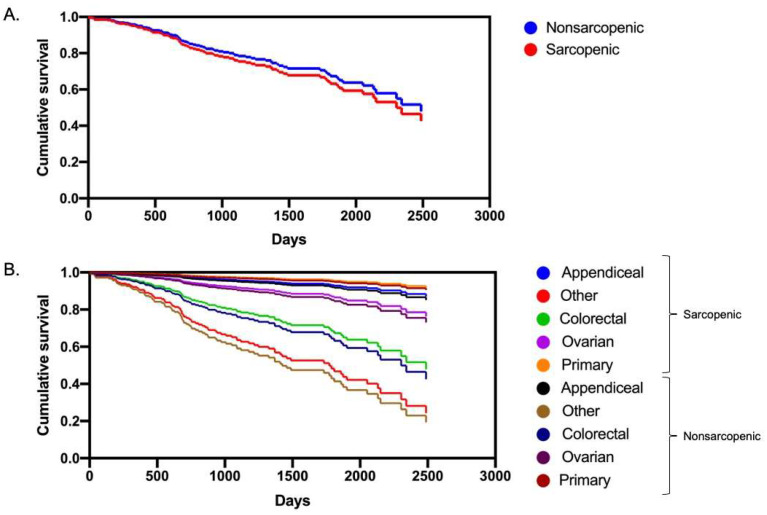
Patient survival according to sarcopenia status in all patients (**A**) and by origin of peritoneal metastases (**B**).

In the multivariate logistic regression analysis of severe complications including sex (female), age, BMI, intraoperative PCI, sarcopenia, and blood loss, only PCI reached statistical significance (OR, 1.05; 95% CI, 1.01 to 1.08, *p* = 0.007) (Table 3). A multivariate linear regression analysis was also performed using CCI as a dependent variable and similar results were obtained. A higher intraoperative PCI was associated with a higher chance of dying (HR, 1.08; 95% CI, 1.05 to 1.12, *p* < 0.0001), while sex (female), age, BMI and sarcopenia had no significant impact on survival (Table 4 and Figure 3A). Kaplan–Meier curves were also generated, with no difference in survival between sarcopenic and nonsarcopenic patients. Median patient follow-up time was 28.0 months [14.0–49.5]. Patients with PM from colorectal origin had worse overall survival in comparison to patients with PM from appendiceal, ovarian or peritoneal origins (Table 4). Sarcopenia did not impact origin-specific overall survival (Figure 3B).

## 4. Discussion

In this largest to date single-institution cohort analyzing the impact of sarcopenia on postoperative and survival outcomes in patients undergoing CRS-HIPEC for PM from various origins, sarcopenia was not found to have a prognostic impact on postoperative complications or on overall survival. Only increasing PCI had a negative impact on complications and survival. Patients with colorectal PM had worse survival outcomes than patients with appendiceal or ovarian metastases and primary peritoneal malignancies.

Thus far, a total of five studies have explored the impact of sarcopenia on postoperative outcomes in patients undergoing CRS-HIPEC [15,16,17,18,19] (Supplementary Table S2). The first study was published in 2015 by Vugt et al. [17]. In their study, using the same cutoffs for defining sarcopenia as in our study, 43.7% of 206 patients included were sarcopenic. More reoperations and severe complications were observed in the sarcopenic patients and sarcopenia was the only variable independently associated with severe complications (OR 0.93; 95% CI 0.87 to 0.99; *p* = 0.018). A year later, Chemama et al. [18] reported similar results to our study, where no differences in complications between sarcopenic and nonsarcopenic patients were noted. However, there were more chemotherapy toxicities in sarcopenic patients (57% vs. 26%, *p* = 0.004), including on multivariable analysis (OR, 3.97; 95% CI 1.52 to 10.39, *p* = 0.005). In our cohort, sarcopenic patients did not have more chemotherapy toxicities. This difference could be possibly explained by how we included all toxicities (i.e., hematologic, metabolic, neurologic, and gastrointestinal toxicities) and not only hematologic ones as reported by Chemama et al. Furthermore, their SMI cutoffs for defining sarcopenia were different. In accordance with our results, Banaste et al. [19] and Galan et al. [15] reported no observable difference in major postoperative complications between sarcopenic and nonsarcopenic patients. However, a more recent study by Agalar et al. [16] revealed that sarcopenic patients had a higher likelihood of morbidity, mortality, and shorter survival time. Interestingly, the proportion of sarcopenic patients affected with colorectal PM in our population was 40.4%, which lies within the range of sarcopenic patients in the aforementioned studies (30.8% and 43.7%) [16,17,18,19].

The conflicting data on sarcopenia and postoperative and oncological outcomes in patients undergoing CRS-HIPEC possibly lies in how complex patients affected with PM are, and many variables should be considered simultaneously when predicting postoperative outcomes. Another important point to raise is the definition of sarcopenia. In this study, sarcopenia was defined according to predetermined cutoff values based on the study by Prado et al. [8]. Their study introduced the concept of sarcopenic obesity, looking at sex-specific cutoffs of SMI associated with survival in cancer patients with BMI ≥ 30 kg/m^2^. The malignancies included were colorectal, respiratory tract, and other less frequent such as anus, pancreas, stomach, and esophagus. The study defined sarcopenia as a SMI < 52.4 cm^2^/m^2^ for men and <38.5 cm^2^/m^2^ for women. Many subsequent studies have used these cutoffs for assessing sarcopenia in surgical patients. It is important to note however that such cutoffs may not be generalizable to other populations, such as nonobese patients included in our cohort. Indeed, the mean BMI in our population was 26.6 kg/m^2^.

Furthermore, other studies assessing the impact of sarcopenia on postoperative outcomes in cancer patients undergoing CRS-HIPEC have used alternative SMI cutoffs such as <41 cm^2^/m^2^ for women and <43 cm^2^/m^2^ for men (if BMI ≤ 24.9 kg/m^2^) or <53 cm^2^/m^2^ for men (if BMI > 25 kg/m^2^) [18] or SMI ≤ 39 cm^2^/m^2^ for women and 55 cm^2^/m^2^ for men [15]. This reflects the lack of consensus on the radiological definition of sarcopenia, which perhaps is an oversimplification of the concept of sarcopenia. Indeed, according to the European Working Group on Sarcopenia in Older People (EWGSOP), sarcopenia is a syndrome with the presence of both low muscle mass and low muscle strength and performance [26]. Our study lacks information on muscle function of patients, which could perhaps be addressed in future prospective studies. Measurement of sarcopenia with CT scans remains an interesting method due to the widespread use of scans in cancer patients and the availability of many softwares for muscle surface calculation. Other surrogate markers for sarcopenia exist and could be obtained through various tools. These include methods to assess for muscle mass, muscle strength and physical performance, such as dual-energy X-ray absorptiometry, handgrip strength or gait speed, respectively [27]. These tools could perhaps offer better prognostic information in a population like ours.

At our institution, the selection of patients for CRS-HIPEC is strict. Referred patients are screened and discussed at multidisciplinary meetings. Despite a possible sarcopenic state in which patients present, an adequate functional status is required prior to undergoing CRS-HIPEC. It is not excluded that sarcopenia could be one of multiple factors affecting postoperative outcomes. However, a good functional status could counterbalance a sarcopenic state, as both entities are not mutually exclusive [28]. Another important factor to consider in our population is total parenteral nutrition (TPN). Our practice involves the initiation of TPN to everyone on as early as postoperative days 1 or 2. Early TPN has been shown to decrease infectious complications in patients undergoing abdominal surgery [29]. It is possible that such a practice contributes to mitigating the detrimental effects of malnutrition in sarcopenic patients. This practice differs from one center to another and may explain why others have found a link between sarcopenia and postoperative complications. Lastly, our institution is a tertiary referral center with expertise in the management of PM. It has been previously demonstrated that morbidity and mortality from CRS-HIPEC is considerably decreased in high-volume expert centers [30]. We therefore believe this too could have explained in part similar the complication rates between sarcopenic and nonsarcopenic patients in our cohort.

The strength of our study lies within the large number of patients evaluated over a long period of time. Previous studies have only evaluated the impact of sarcopenia on patients undergoing CRS-HIPEC for PM from colorectal [16,17,18,19] or appendiceal and primary [15] origins. We decided to include different origins for two reasons: first, all patients diagnosed with PM are at risk of cachexia and sarcopenia. We did not want to limit our analysis to one subcategory of patients. Second, to our knowledge, no study thus far has evaluated the impact of sarcopenia on patients undergoing CRS-HIPEC for PM from various origins, especially gynecological malignancies such as in our study.

This study is limited by its retrospective nature. Several factors could have contributed to selection bias. First, some patients were excluded from the study based on missing preoperative CT scans. Second, because many patients are referred to our institution by other surgeons and that potential candidates for CRS-HIPEC are closely evaluated prior to considering any invasive intervention, some sarcopenic patients may have been excluded from our analysis because they were considered unfit for surgery. Given the retrospective nature of this study, convenience sampling was opted for patient selection. However, our sample size may have not been adequately large to detect as difference between sarcopenic and nonsarcopenic patients.

## 5. Conclusions

In conclusion, CT-measured sarcopenia in our cohort was not associated with worse rates of postoperative severe complications or worse survival rates. Given the high morbidity of CRS-HIPEC procedures, research aiming at reducing complications merit our attention. Future prospective trials assessing the prognostic value sarcopenia in patients affected with PM are necessary. If sarcopenia truly represents a significant predictor of postoperative outcomes, it could be used in preoperative risk assessment. Sarcopenic patients awaiting CRS-HIPEC could undergo prehabilitation to potentially enhance recovery and quality of life after surgery.

## Figures and Tables

**Table 1:** Patient Baseline Demographic and Clinical Characteristics.

Variable	All (*n* = 312)	Sarcopenia (*n* = 88)	No Sarcopenia (*n* = 224)	*p* Value
Mean age at time of surgery, years (SD)	57.6 (10.3)	58.1 (10.7)	57.5 (10.2)	0.63
(min–max)	(21.7–79.3)	(21.7–73.7)	(24.7–79.3)	
Male sex, *n* (%)	107 (34.3)	41 (46.6)	66 (29.5)	0.0053
Mean delay from scan to surgery, days (SD)	44.2 (37.0)	42.1 (28.6)	45.0 (39.9)	0.53
(min, max)	(1–245)	(1–122)	(1–245)	
Mean BMI, kg/m^2^ (SD)	26.6 (5.5)	23.6 (3.9)	27.7 (5.7)	<0.0001
(min–max)	(15.5–46.9)	(20.7–25.5)	(17.5–46.9)	
Mean SMI, cm^2^/m^2^ (SD)	48.7 (10.3)	41.4 (7.3)	51.2 (9.8)	<0.0001
(min–max)	(30.0–81.3)	(30.0–75.1)	(38.5–81.3)	
Origin of PC, *n* (%)				0.43
Colorectal	126 (40.4)	37 (42.0)	89 (39.7)	
Appendix	88 (28.2)	27 (30.7)	61 (27.2)	
Ovarian	66 (21.2)	15 (17.0)	51 (22.8)	
Peritoneal	24 (7.7)	5 (5.7)	19 (8.5)	
Other *	8 (2.6)	4 (4.5)	4 (1.8)	
Timing of PC, *n* (%)				0.45
Synchronous	168 (53.9)	52 (59.0)	116 (51.8)	
Metachronous	120 (38.5)	31 (35.2)	89 (39.7)	
Primary	24 (7.7)	5 (5.7)	19 (8.5)	

* Other: endometrium (3), stomach (3), small bowel (1), anus (1) BMI = body mass index; SMI = skeletal muscle index; PC = peritoneal carcinomatosis.

**Table 2:** Intra- and Postoperative Outcomes.

Variable	All (*n* = 312)	Sarcopenia (*n* = 88)	No Sarcopenia (*n* = 224)	*p* Value
Mean operative time, min (SD)	423.8 (118.8)	422.1 (128.8)	424.5 (114.6)	0.87
(min–max)	(100–870)	(180–840)	(100–870)	
Mean estimated blood loss, ml (SD)	877.1 (739.2)	929.3 (909.9)	856.6 (661.5)	0.44
(min–max)	(50–4800)	(50–4800)	(50–4000)	
CC score				0.059
0	273 (87.5%)	82 (93.2%)	191 (85.3%)	
1–2	39 (12.5%)	6 (6.8%)	33 (14.7%)	
Mean intraoperative PCI (SD)	12.0 (8.9)	11.2 (9.0)	12.3 (8.8)	0.34
(min–max)	(0–39)	(0–39)	(0–39)	
Clavien-Dindo ≥ III, *n* (%)	66 (21.2%)	14 (15.9%)	52 (23.2%)	0.17
Median CCI score [IQR]	20.9 [30.8]	20.9 [29.6]	20.9 [32.0]	0.47
(min–max)	(0–100)	(0–100)	(0–100)	
HIPEC-related toxicity, *n* (%)	37 (11.9%)	9 (10.2%)	28 (12.5%)	0.70
Mean length of stay, days (SD)	17.4 (10.9)	17.9 (12.3)	17.2 (10.3)	0.60
(min–max)	(3–80)	(3–80)	(5–65)	
Mean duration of parenteral nutrition, days (SD)	13.5 (11.7)	11.3 (7.1)	11.1 (7.6)	0.83
(min–max)	(2–60)	(2–51)	(3–60)	
Death (Clavien-Dindo V), *n* (%)	3 (1.0%)	1 (1.1%)	2 (0.9%)	0.84

CC = completeness of cytoreduction; PCI = peritoneal carcinomatosis index; HIPEC = hyperthermic intraperitoneal chemotherapy; CCI = comprehensive complication index.

**Table 3:** Multivariate Regression Evaluating 90-day Severe Complications.

Variable	Odds Ratio	95% CI	*p* Value
Sex (female)	1.10	0.59–2.06	0.77
Age (years)	1.02	0.99–1.05	0.29
BMI (kg/m^2^)	1.02	0.97–1.08	0.42
Intraoperative PCI	1.05	1.01–1.08	0.007
Sarcopenia	0.70	0.33–1.41	0.33
Blood loss	1.00	0.99–1.00	0.59

BMI = body mass index; PCI = peritoneal carcinomatosis index.

**Table 4:** Patient Survival (multivariate COX regression).

Variable	Hazard Ratio	95% CI	*p* Value
Sex (female)	0.78	0.46–1.34	0.37
Age (years)	1.00	0.98–1.03	0.77
BMI (kg/m^2^)	0.99	0.95–1.04	0.82
Intraoperative PCI	1.08	1.05–1.12	<0.0001
Sarcopenia	1.17	0.68–2.0	0.57
Origin of PC			
Colorectal vs. appendix	0.19	0.09–0.37	<0.0001
Colorectal vs. ovarian	0.37	0.18–0.77	<0.0001
Colorectal vs. peritoneal	0.12	0.04–0.32	0.0090
Colorectal vs. other *	1.93	0.64–4.75	0.77

* Other: endometrium (3), stomach (3), small bowel (1), anus (1) BMI = body mass index; PCI = peritoneal carcinomatosis index; PC = peritoneal carcinomatosis.

## Data Availability

The data presented in this study are available on request from the corresponding author.

## Supplementary Materials

The following supporting information can be downloaded at: https://www.mdpi.com/article/10.3390/curroncol29120730/s1, Table S1: Postoperative Outcomes Stratified by Sarcopenic State and PM Origin; Table S2: A review of studies assessing the impact of sarcopenia on postoperative outcomes in cancer patients undergoing CRS-HIPEC [15,16,17,18,19].
