# Supplementary material for: The Impact of Computed Tomography Measurements of Sarcopenia on Postoperative and Oncologic Outcomes in Patients Undergoing Cytoreductive Surgery and Hyperthermic Intraperitoneal Chemotherapy
Source: Curr Oncol. 2022 Nov 29;29(12):9314–24. doi: 10.3390/curroncol29120730 (PMC9777197; doi:10.3390/curroncol29120730)
Supplement: Supplementary file 1 [file curroncol-29-00730-s001.zip › curroncol-2038790-supplementary.pdf]

**Table S1.** Postoperative Outcomes Stratified by Sarcopenic State and PM Origin

| Complications | All patients | Sarcopenic | Nonsarcopenic | *P* value |
|---|---|---|---|---|
| Colorectal origin | | | | |
| | n = 126 | n = 37 | n = 89 | |
| Clavien-Dindo ⩾ III, n (%) | 23 (18.3) | 5 (13.5) | 18 (20.2) | 0.45 |
| Median CCI score [IQR]<br>(min - max) | 20.9 [29.6]<br>(0 – 100) | 20.9 [29.6]<br>(0 – 52.6) | 20.9 [30.8]<br>(0 – 100) | 0.80 |
| Appendiceal origin | | | | |
| | n = 88 | n = 27 | n = 61 | |
| Clavien-Dindo ⩾ III, n (%) | 24 (27.2) | 6 (22.2) | 18 (29.5) | 0.48 |
| Median CCI score [IQR]<br>(min - max) | 20.9 (33.3)<br>(0 – 100) | 20.9 [25.3]<br>(0 – 100) | 22.6 [46.2]<br>(0 – 100) | 0.42 |
| Ovarian origin | | | | |
| | n = 66 | n = 15 | n = 51 | |
| Clavien-Dindo ⩾ III, n (%) | 15 (22.7) | 2 (13.3) | 13 (25.5) | 0.92 |
| Median CCI score [IQR]<br>(min - max) | 20.9 [29.5]<br>(0 – 71.4) | 20.9 [21.5]<br>(0 – 59.9) | 20.9 [32.8]<br>(0 – 71.4) | 0.78 |
| Peritoneal origin | | | | |
| | n = 24 | n = 5 | n = 19 | |
| Clavien-Dindo ⩾ III, n (%) | 3 (12.5) | 0 (0) | 3 (15.8) | 1.00 |
| Median CCI score [IQR]<br>(min - max) | 20.9 [16.5]<br>(0 – 54.1) | 20.9 [12.2]<br>(0 – 24.2) | 20.9 [23.0]<br>(0 – 54.1) | 0.67 |
| Other origin* | | | | |
| | n = 8 | n = 4 | n = 4 | |
| Clavien-Dindo ⩾ III, n (%) | 1 (12.5) | 1 (25) | 0 (0) | 1.00 |
| Median CCI score [IQR]<br>(min - max) | 0 (20.9)<br>(0 – 34.6) | 0 [8.7]<br>(0 – 34.6) | 10.5 [20.9]<br>(0 – 20.9) | 0.74 |

PM = peritoneal metastasis; CCI = comprehensive complication index

*Other origin: endometrium (3), stomach (3), small bowel (1), anus (1)

**Table S2.** A review of studies assessing the impact of sarcopenia on postoperative outcomes in cancer patients undergoing CRS-HIPEC.

| Author | Year | Nature of study | Patients | Origin | Sarcopenia measurement | Outcomes |
|---|---|---|---|---|---|---|
| Vugt [17] | 2015 | Retrospective | n = 206<br><br>90 (43.7%) with sarcopenia | Colorectal cancer (CRC) | CT – L3 level<br><br>SMI cut-off for sarcopenia: 52.4 $cm^2/m^2$ for men and 38.5 $cm^2/m^2$ for women | More reoperations in sarcopenic patients (25.6 vs. 12.1%; p = 0.012)<br><br>Lower muscle index in patients with severe complications (85.6 vs. 110.2 $cm^2/m^2$; p = 0.008)<br><br>Multivariable analysis revealed that sarcopenia is an independent risk factor for complications (OR = 0.93; p = 0.018) |
| Chemama [18] | 2016 | Outcomes were prospectively recorded within 30 days post-op<br><br>Sarcopenia was retrospectively measured | n = 97<br><br>39 (40%) patients with sarcopenia | CRC | CT – L3 level within 2 months of surgery<br><br>SMI cut-off: <41 $cm^2/m^2$ for women and <43 $cm^2/m^2$ if BMI ⩽24.9 $kg/m^2$ and <53 $cm^2/m^2$ if BMI >25 $kg/m^2$ for men | More chemotherapy toxicities in sarcopenic patients (57 vs. 26%; p = 0.004)<br><br>No difference in complications between sarcopenic and nonsarcopenic patients<br><br>Multivariable analysis revealed that sarcopenia is an independent risk factor for chemotherapy toxicity (OR = 3.97; p = 0.005) |
| Banaste [19] | 2017 | Prospective | n = 214<br><br>90 (42%) were sarcopenic | CRC | CT – L3 level 2 days before surgery | Primary aim of this study was to assess albumin levels with outcomes<br><br>In univariate analysis, median survival in sarcopenic arm was not different than survival in nonsarcopenic arm |

| | | | | | | |
|---|---|---|---|---|---|---|
| | | | | | SMI cut-off: 52.4 $cm^2/m^2$ for men and 38.5 $cm^2/m^2$ for women | Sarcopenia was not associated with increased postoperative major complications or poor long-term prognosis |
| Galan [15] | 2018 | Retrospective | n = 115<br><br>82 treated for PMP and 33 for PM<br>64 (55.7%) were sarcopenic | PMP<br><br>PM | CT – L3 level<br><br>SMI: $\leqslant$39 $cm^2/m^2$ for women and 55 $cm^2/m^2$ for men | Major postoperative complications occurred in 63 patients (54.8%), without observable difference between sarcopenic and non-sarcopenic patients (56.2% vs. 52.9%; p = 0.723).<br><br>The median OS was 73.3 for the patients with a normal muscle mass and 57.2 months for the sarcopenic patients (p = 0.05) → independent predictive factor for OS (no multivariable analysis though) |
| Agalar [16] | 2020 | Retrospective (longitudinal cohort study) | n = 65<br><br>30.8% were sarcopenic | CRC | CT – L3 level<br><br>SMI: 52.4 $cm^2/m^2$ for men and 38.5 $cm^2/m^2$ for women | Presence of sarcopenia was associated with older age (59.6 (9.2) vs. 52.1 (14.4) years; p = 0.038), higher likelihood of morbidity (70.0% vs. 35.6%; p = 0.015) and mortality (90.0% vs. 55.6%; p = 0.010) and shorter survival time (17.7 vs. 37.9 months; p = 0.005).<br><br>Cox regression analysis revealed that the presence of sarcopenia (HR 2.245, 95% CI 0.996–5.067, p = 0.050) was a significant predictor of increased likelihood of mortality. |

CRS-HIPEC = cytoreductive surgery with hyperthermic intraperitoneal chemotherapy; SMI = skeletal muscle index; CT = computed tomographic; L3 = lumbar level 3; CRC = colorectal cancer; PMP = pseudomyxoma peritonei; PM = peritoneal mesothelioma

## References

[17] van Vugt, J.L.A.; Braam, H.J.; van Oudheusden, T.R.; Vestering, A.; Bollen, T.L.; Wiezer, M.J.; de Hingh, I.H.J.T.; van Ramshorst, B.; Boerma, D. Skeletal Muscle Depletion is Associated with Severe Postoperative Complications in Patients Undergoing Cytoreductive Surgery with Hyperthermic Intraperitoneal Chemotherapy for Peritoneal Carcinomatosis of Colorectal Cancer. *Ann. Surg. Oncol.* **2015**, *22*, 3625–3631. https://doi.org/10.1245/s10434-015-4429-z.

[18] Chemama, S.; Bayar, M.A.; Lanoy, E.; Ammari, S.; Stoclin, A.; Goéré, D.; Elias, D.; Raynard, B.; Antoun, S. Sarcopenia is Associated with Chemotherapy Toxicity in Patients Undergoing Cytoreductive Surgery with Hyperthermic Intraperitoneal Chemotherapy for Peritoneal Carcinomatosis from Colorectal Cancer. *Ann. Surg. Oncol.* **2016**, *23*, 3891–3898. https://doi.org/10.1245/s10434-016-5360-7.

[19] Banaste, N.; Rousset, P.; Mercier, F.; Rieussec, C.; Valette, P.-J.; Glehen, O.; Passot, G.; Preoperative nutritional risk assessment in patients undergoing cytoreductive surgery plus hyperthermic intraperitoneal chemotherapy for colorectal carcinomatosis. *Int. J. Hyperther.* **2017**, *34*, 589–594. https://doi.org/10.1080/02656736.2017.1371342.

[15] Galan, A.; Rousset, P.; Mercier, F.; Képénékian, V.; Valette, P.-J.; Glehen, O.; Passot, G. Overall survival of pseudomyxoma peritonei and peritoneal mesothelioma patients after cytoreductive surgery and hyperthermic intraperitoneal chemotherapy can be predicted by computed tomography quantified sarcopenia. *Eur. J. Surg. Oncol.* **2018**, *44*, 1818–1823. https://doi.org/10.1016/j.ejso.2018.07.060.

[16] Agalar, C.; Sokmen, S.; Arslan, C.; Altay, C.; Basara, I.; Canda, A.E.; Obuz, F. The impact of sarcopenia on morbidity and long-term survival among patients with peritoneal metastases of colorectal origin treated with cytoreductive surgery and hyperthermic intraperitoneal chemotherapy: A 10-year longitudinal analysis of a single-center experience. *Tech. Coloproctol.* **2020**, *24*, 301–308. https://doi.org/10.1007/s10151-020-02159-z.
